# Ecophysiological Keys to the Success of a Native-Expansive Mediterranean Species in Threatened Coastal Dune Habitats

**DOI:** 10.3390/plants14152342

**Published:** 2025-07-29

**Authors:** Mario Fernández-Martínez, Carmen Jiménez-Carrasco, Mari Cruz Díaz Barradas, Juan B. Gallego-Fernández, María Zunzunegui

**Affiliations:** Departamento de Biología Vegetal y Ecología, Facultad de Biología, Apartado 1095, 41080 Sevilla, Spain; mfernandez27@us.es (M.F.-M.); carmenjimenezcarrasco1@gmail.com (C.J.-C.); diaz@us.es (M.C.D.B.); galfer@us.es (J.B.G.-F.)

**Keywords:** invasive behaviour, *Juniperus phoenicea*, *Juniperus macrocarpa*, photochemical efficiency, *Pinus pinea*, *Retama monosperma*, shoot water potential, stable isotope

## Abstract

Range-expanding species, or neonatives, are native plants that spread beyond their original range due to recent climate or human-induced environmental changes. *Retama monosperma* was initially planted near the Guadalquivir estuary for dune stabilisation. However, changes in the sedimentary regime and animal-mediated dispersal have facilitated its exponential expansion, threatening endemic species and critical dune habitats. The main objective of this study was to identify the key functional traits that may explain the competitive advantage and rapid spread of *R. monosperma* in coastal dune ecosystems. We compared its seasonal responses with those of three co-occurring woody species, two native (*Juniperus phoenicea* and *J. macrocarpa*) and one naturalised (*Pinus pinea*), at two sites differing in groundwater availability within a coastal dune area (Doñana National Park, Spain). We measured water relations, leaf traits, stomatal conductance, photochemical efficiency, stable isotopes, and shoot elongation in 12 individuals per species. Repeated-measures ANOVA showed significant effects of species and species × season interaction for relative water content, shoot elongation, effective photochemical efficiency, and stable isotopes. *R. monosperma* showed significantly higher shoot elongation, relative water content, and photochemical efficiency in summer compared with the other species. Stable isotope data confirmed its nitrogen-fixing capacity. This characteristic, along with the higher seasonal plasticity, contributes to its competitive advantage. Given the ecological fragility of coastal dunes, understanding the functional traits favouring the success of neonatives such as *R. monosperma* is essential for biodiversity conservation and ecosystem management.

## 1. Introduction

Species coexistence within ecological communities is influenced by a complex interplay of abiotic and biotic factors [1]. However, in recent decades, global climate change and human-induced environmental alterations have modified these dynamics, enabling many plant species to expand their range beyond their historical distribution ranges [2,3]. While some of these species integrate into their new environments with minimal ecological impact, others exhibit rapid expansion, outcompeting the native flora and potentially altering community composition and ecosystem functioning [4]. In some cases, anthropogenic changes in the environment may directly favour the success of the expansion of certain native or alien species [5].

Among these expanding species, it is important to distinguish between invasive alien species and native range-expanding species [6,7]. Invasive alien species are those transported, either intentionally or unintentionally, by human activities beyond their native distribution ranges to areas they cannot reach by natural dispersal mechanisms [8]. Once established in a new zone, they can outcompete native flora, often leading to biodiversity loss [9], severe modifications in ecosystem properties [10], and homogenisation of species pools across habitats in the recipient region [11,12]. In contrast, native range-expanding species, also referred to as expansive species or neonatives, are native species that expand their range in response to environmental changes, such as climate warming or land use alterations [6]. By definition, expansive species are those that increase their distribution and colonise new habitats within their native range [2,6]. These species can significantly influence ecosystem dynamics, particularly in sensitive habitats such as coastal dune systems.

While the ecological impacts of invasive alien species are well documented, much less is known about the potential consequences of native range-expanding species on community structure and ecosystem functioning. Understanding whether these neonatives share traits similar to those of invasive ones is essential, especially in ecosystems already under stress from anthropogenic change.

Due to human activity, environmental disturbances are increasingly altering ecosystems through processes such as climate change, land use transformation, pollution, or eutrophication. These changes can lead to the local extinction of native species and a reduction in biodiversity [13,14,15]. At the same time, they may also facilitate the expansion of certain native species beyond their historical distribution areas [16,17,18].

Coastal dunes are dynamic and heterogeneous ecosystems that lie at the interface between the sea and land [19]. These habitats play a crucial ecological role as natural barriers against sea-level rise and storm surges, stabilising sediments and protecting inland ecosystems from erosion and saltwater intrusion [20]. Moreover, they support unique and specialised vegetation adapted to harsh conditions such as salt spray, sand movement, poor soils, and drought [21,22]. In fact, plants in many coastal dune systems experience arid-like conditions due to the low water retention capacity and high hydraulic conductivity of sandy soils. The macropores, typical of sands, lead to rapid drainage, making plant survival challenging, even when rainfall is not limited [23]. Despite these constraints, coastal dunes are characterised by particularly high plant species richness, including numerous endemic species and a wide array of functional types [22,24,25]. Beyond their ecological value, these ecosystems provide essential ecosystem services such as environmental quality, support for local economies, and landscape aesthetics [20,26,27].

However, coastal dunes with low vegetation cover may be particularly vulnerable to colonisation by invasive or expansive species due to reduced competition. Moreover, human activities have destroyed coastal ecosystems, producing habitat fragmentation, modifying their dynamics, and consequently causing biodiversity loss [28]. In this context, several ecological hypotheses have been proposed to explain species success under stress or low resistance, including theories that link plant colonisation to environmental harshness or species richness [29,30,31]. While such frameworks may help contextualise patterns of colonisation in dune systems, the specific mechanisms underpinning the success of many expanding species remain poorly understood.

*Retama monosperma* (L.) Boiss is a leguminous shrub native to the Mediterranean coastal sandy environments of the SW of the Iberian Peninsula. It has been described as an exotic invader in California [32] and Australia [33]. In addition, this species has undergone rapid expansion within its native range, particularly in the dune systems of Doñana, near the Guadalquivir estuary, and in other coastal areas of the SW of the Iberian Peninsula [34,35]. Initially introduced for dune stabilisation, its proliferation has been facilitated by changes in sediment dynamics and seed-dispersal animals [35]. This expansion poses a potential threat to endemic species and the integrity of these ecosystems, raising concerns about its ecological impact [36,37,38]. However, the mechanisms underlying the success of *R. monosperma* in these environments remain poorly understood.

Several authors have described how these species can exert facilitation effects on their environments [39], yet these traits may also contribute to their expanding capacity [35,38]. A key factor that potentially underlies the success of *R. monosperma* is its morphophysiological adaptability [40]. Traits such as water-use efficiency [36], growth dynamics [35,37], and the ability to fix atmospheric nitrogen [41] may confer a competitive advantage this species in the resource-limited environment of coastal dunes [41].

Mediterranean plants typically exhibit a set of morphophysiological traits that enable them to tolerate and withstand summer drought [42,43,44,45], and these harsh conditions are often further enhanced in coastal dune habitats. We suggest that the analysis of morphophysiological traits and their seasonal variability could be important for assessing the expanding capacity of different species in coastal dunes. Previous studies have highlighted the role of such traits in the success of invasive species such as *Acacia longifolia* [46] or *Oenothera drummondii* [47]. Therefore, understanding the physiological mechanisms that allow certain species to establish and spread successfully, both within and beyond their historical distribution areas, may be essential for predicting future shifts in vegetation composition and developing effective management strategies.

To investigate these questions, we conducted a comparative study assessing the seasonal physiological responses of *R. monosperma* and three co-occurring woody species representative of different functional strategies in the coastal dune system: *Juniperus phoenicea*, *J. macrocarpa* (native, slow-growing conifers), and *Pinus pinea* (a long-lived, naturalised conifer widely used in dune stabilisation) [48,49]. The study was carried out at two different levels of groundwater availability. The main objective was to determine whether the ecophysiological traits of *R. monosperma* provide a growth advantage over the co-occurring species, potentially facilitating its rapid expansion in dune ecosystems. By identifying the functional traits that contribute to its success, this research provides valuable insights into the mechanisms driving plant expansion in coastal dunes and offers a scientific basis for developing effective management strategies.

We hypothesised that the morphophysiological traits of *R. monosperma* provide a competitive advantage over native dune species, facilitating its expansion. Specifically, we predict that *R. monosperma* will (i) exhibit higher shoot elongation rates and (ii) maintain greater leaf water content and higher photochemical efficiency during summer, indicating enhanced drought tolerance. In addition, we confirmed its nitrogen-fixing capacity through stable isotope analysis, a trait known to be advantageous in nutrient-poor, sandy soils.

## 2. Results

**Figure 1:**
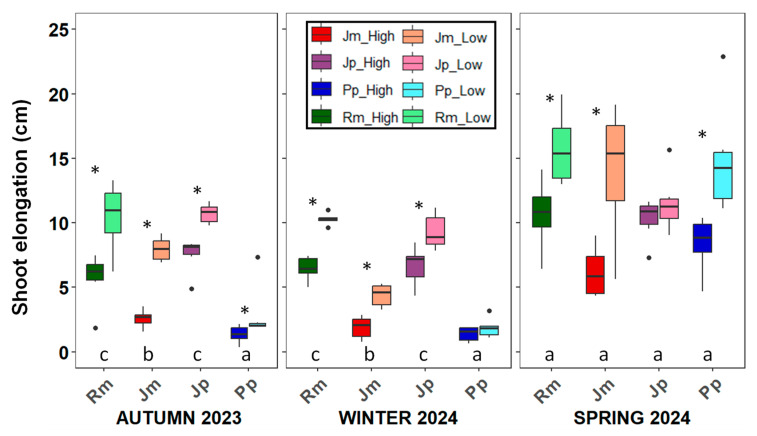
Seasonal shoot elongation (mean ± SD) for the four study species growing in the low and high zones during the growing season from autumn to spring. Boxplots show the median, interquartile range, and potential outliers. Asterisks indicate significant differences between the low and high zones (*p* < 0.05). Different letters denote significant differences among species within each season according to Tukey’s post hoc test. Rm: *Retama monosperma*; Jm: *Juniperus macrocarpa*; Jp: *J. phoenicea*; Pp: *Pinus pinea*.

Seasonal shoot growth showed interspecific variation and a clear influence of the topographic position (Table A1). Across all species, growth initiated slowly in autumn and increased markedly during spring, coinciding with the more favourable environmental conditions (Figure 1). *R. monosperma* showed the highest shoot elongation at the onset of the growth period, while *P. pinea* consistently exhibited the lowest values. Both *J. phoenicea* and *J. macrocarpa* had intermediate growth rates. Peak elongation occurred in spring for all species when no significant differences among species were detected. A consistent pattern was observed across species: individuals located in the low site showed greater shoot elongation than those growing in the upper dune areas. This suggests that small topographic variations in water or nutrient availability may modulate growth responses across the dune gradient, regardless of species identity.

**Figure 2:**
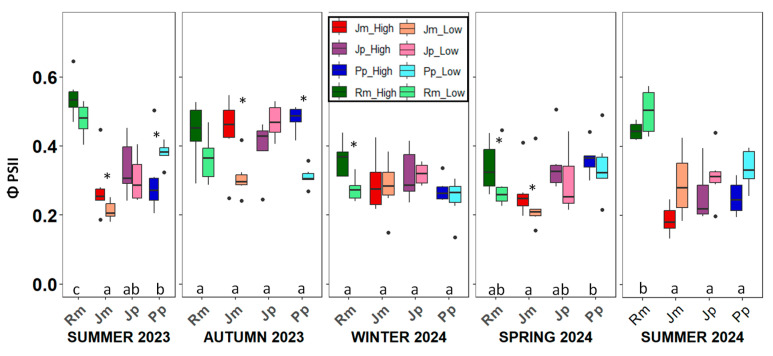
Seasonal variation in the effective photochemical efficiency (Φ_PSII_) across the five sampling campaigns for the four study species. Boxplots show the median, interquartile range, and potential outliers. Asterisks indicate significant differences between the low and high zones (*p* < 0.05). Different letters denote significant differences among species within each season according to Tukey’s post hoc test. Rm: *Retama monosperma*; Jm: *Juniperus macrocarpa*; Jp: *J. phoenicea*; Pp: *Pinus pinea*.

Effective photochemical efficiency showed significant variation among species and across seasons, but no significant differences were found between the high and low zones (Table A1). During the wet seasons, there were no significant interspecific differences (except for *J. phoenicea* in spring); however, in the two summer campaigns, *R. monosperma* consistently exhibited the highest photochemical efficiency, with values around 0.5—surpassing not only the other species (which averaged 0.3 in summer), but also its own winter, spring, and autumn (0.35, 0.35, and 0.4, respectively) values (Figure 2).

**Figure 3:**
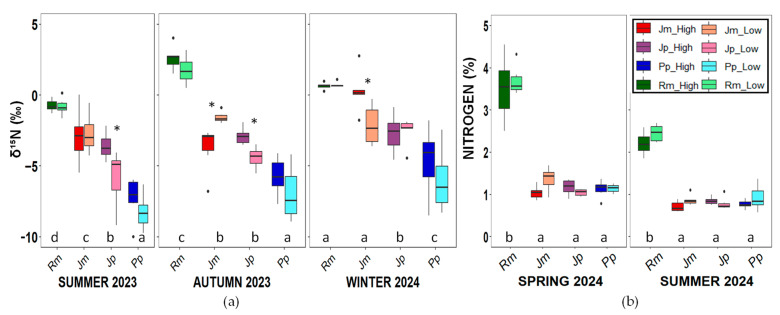
(**a**) Nitrogen isotopic composition (δ^15^N) and (**b**) foliar nitrogen content (%N) for the four studied species in the low and high zones. Boxplots show the median, interquartile range, and potential outliers. Asterisks indicate significant differences between zones (*p* < 0.05). Letters indicate significant differences among species and zones according to Tukey’s and Kruskal-Wallis tests (*p* < 0.05). Rm: *Retama monosperma*; Jm: *Juniperus macrocarpa*; Jp: *J. phoenicea*; Pp: *Pinus pinea*.

**Figure 4:**
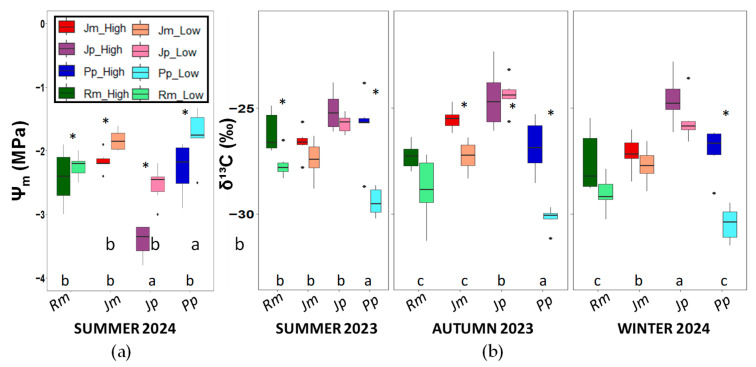
(**a**) Shoot water potential (Ψ_m_) measured during the summer campaign in the four study species and (**b**) carbon isotopic composition (δ^13^C) as a proxy for long-term intrinsic water-use efficiency. Boxplots show the median, interquartile range, and potential outliers. Asterisks indicate significant differences between zones (*p* < 0.05). Different letters denote significant differences among species within each season according to Tukey’s post-hoc test (*p* < 0.05). Rm: *Retama monosperma*; Jm: *Juniperus macrocarpa*; Jp: *J. phoenicea*; Pp: *Pinus pinea*.

These patterns were consistent across traits: the species with the highest shoot elongation and effective photochemical efficiency, *R. monosperma*, also showed the highest foliar nitrogen content and δ^15^N values indicative of biological nitrogen fixation (Figure 3 and Figure 4). Foliar nitrogen content was similar across species and zones, except for *R. monosperma*, which exhibited significantly higher values (3.5 ± 0.5%) compared with the other three species (Table A2), whose average values were poor, at around 0.8%. This pattern aligns with the δ^15^N isotopic signatures, which show marked interspecific differences. *R. monosperma* displayed δ^15^N values close to 0 ± 1‰, consistent with biological nitrogen fixation. In contrast, *P*. *pinea* exhibited the most negative δ^15^N values, ranging from −7 to −5‰, typical of species relying exclusively on mineralised, soil-derived nitrogen in nutrient-poor environments. The two Juniperus species showed intermediate δ^15^N values, from 5 to 2.5‰. These significant interspecific differences suggest that the coexisting species employ contrasting nitrogen acquisition strategies.

Shoot water potential was measured only during the summer campaign. Significant differences were found between the two zones, with more negative values recorded in the upper zone compared to the lower zone for all species except Retama. The species experiencing the highest water stress was *J. phoenicea*, which reached values of around −4.0 ± 0.98 MPa in the upper zone. The remaining species did not show significant differences among them, except for *P. pinea* from the low zone.

The statistically significant contrasting patterns observed in δ^13^C between *J. phoenicea* and *P. pinea* highlight the interspecific differences in water-use strategies. *J. phoenicea* exhibited the least negative δ^13^C values, indicating a higher WUEi and stronger stomatal control. This was consistent with its more negative Ψm values in summer, suggesting greater exposure to water stress and a conservative water-use strategy under dry conditions. In contrast, *P. pinea* showed the most negative δ^13^C values and the least negative Ψm, indicating lower WUEi and a more open stomatal behaviour. This pattern suggests a less conservative strategy, with greater water loss through transpiration, compared with *J. phoenicea*. *R. monosperma* and *J. macrocarpa* displayed intermediate shoot water potential and δ^13^C values, suggesting moderate stomatal regulation and water stress levels compared to the other two species.

**Figure 5:**
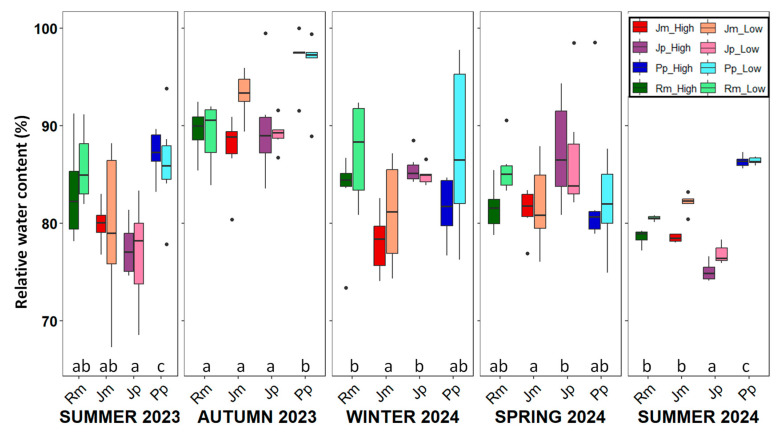
Seasonal variation in relative water content (%) across the five sampling campaigns for the four species under study. Boxplots show the median, interquartile range, and potential outliers. Asterisks indicate significant differences between the low and high zones (*p* < 0.05). Different letters denote significant differences among species within each season, according to Tukey’s post-hoc test. (*p* < 0.05). Rm: *Retama monosperma*; Jm: *Juniperus macrocarpa*; Jp: *J. phoenicea*; Pp: *Pinus pinea*.

RWC exhibited a seasonal pattern consistent with Ψm and WUEi, with the lowest values observed in summer (Figure 5). Significant differences were detected among species during this season, with *P.* pinea showing the highest RWC, indicative of a better water status, while *J. phoenicea* displayed the lowest RWC, reflecting greater water stress. These findings further support species-specific water-use strategies under summer drought conditions.

**Figure 6:**
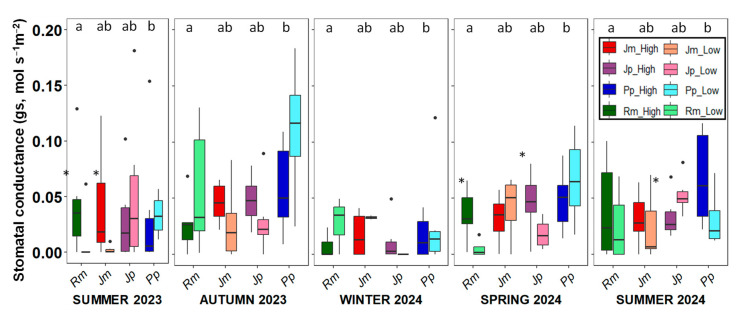
Seasonal variation in stomatal conductance across the five sampling campaigns for the four study species. Boxplots show the median, interquartile range, and potential outliers. Asterisks indicate significant differences between the low and high zones (*p* < 0.05). Different letters denote significant differences among species within each season according to Tukey’s post hoc test. (*p* < 0.05). Rm: *Retama monosperma*; Jm: *Juniperus macrocarpa*; Jp: *J. phoenicea*; Pp: *Pinus pinea*.

Stomatal conductance was consistently higher in *P. pinea* than in the other species across the sampling periods, reflecting its distinctive water-use strategy (Figure 6). This higher conductance aligns with the observed higher RWC, less negative Ψ_m_ during summer, and most negative δ^13^C values, indicating lower water stress and less conservative stomatal control. In contrast, no significant differences in g_s_ were found among *R. monosperma*, *J. phoenicea*, and *J*. *macrocarpa*. No clear seasonal pattern was observed in stomatal conductance.

LAI, LMA, and LDMC varied significantly among seasons and species (Table A1). In general, LAI peaked in spring for all species, with *J*. *phoenicea* and *J. macrocarpa* showing the highest values (6.3 ± 1.8 1 m^2^ m^−2^ and 6.7 ± 2.1 m^2^ m^−2^, respectively), reflecting a denser canopy during the main growth period (Table A3). The lowest LAI values were in winter, particularly for *P. pinea* (0.9–1.11 m^2^ m^−2^), which consistently showed the lowest LAI throughout the year. LDMC and LMA showed parallel seasonal patterns (Table A3). *R. monosperma* consistently displayed the highest LMA and LDMC values across all seasons, with LMA reaching 474 ± 78 g m^−2^ and LDM 544 ± 28.6 mg g^−1^ in September. These elevated values are due to the presence of cladodes (modified stems), which are thicker and denser than the foliage leaves. By contrast, *P. pinea* showed the lowest LMA and LDMC values, especially in winter (173 ± 34 g/m^2^), indicating thinner and less dense tissues. These results suggest that *R. monosperma* and *J. phoenicea* develop tougher, denser leaves compared with *P. pinea*, especially during summer, which could contribute to their greater drought resistance.

**Figure 7:**
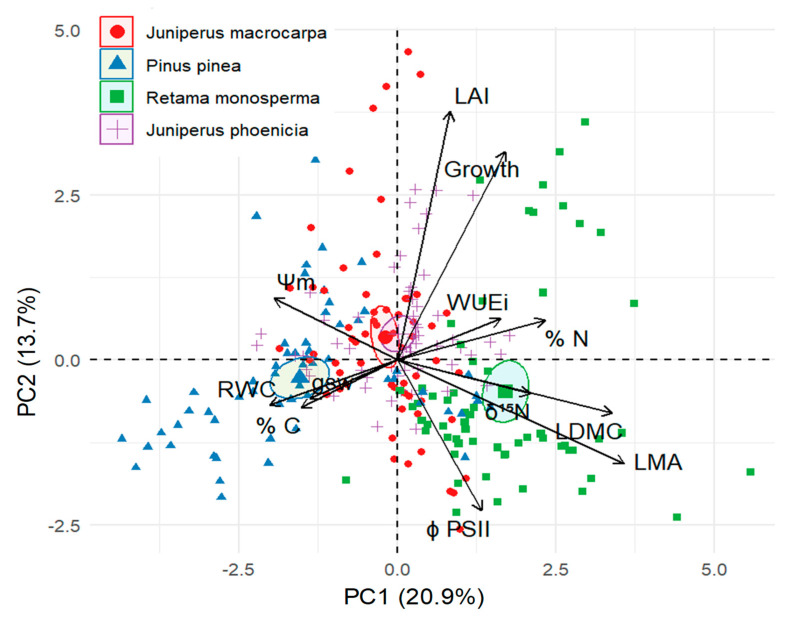
Principal component ordination biplot of morpho-physiological traits measured in the four study species (*Pinus pinea*, *Juniperus macrocarpa*, *J. phoenicea*, and *Retama monosperma*) across the wet and dry seasons. Arrows indicate the direction and strength (loading) of the variables contributing to each axis. Species are represented by ellipses encompassing 95% confidence intervals around the group centroids. Abbreviations of variables and loading factors are shown in Table A4).

PCA of the morphophysiological traits across seasons and species revealed two principal components with eigenvalues, jointly explaining 34.6% of the total variance (Figure 7). The first axis (PC1), which accounted for 20.9% of the variance, was primarily associated with LDMC, LMA, %N, δ^15^N, RWC and Ψ_m_ (loading factors: 0.76, 0.72, and 0.51, 0.45, 0.43, 0.42, respectively; see Table A3). This axis represents an integrative gradient of resource-use strategies across species. *Retama monosperma* was separated at the positive end of PC1, characterised by high foliar %N, largely derived from biological nitrogen fixation, and for higher LMA of its cladodes. In contrast, *Pinus pinea* occupied the negative end of PC1, associated with better water status, as indicated by higher RWC and Ψ_m_ (Figure 4 and Figure 5). The two Juniperus species (*J. macrocarpa* and *J. phoenicea*) occupied intermediate positions along PC1, suggesting more conservative and balanced trait combinations with moderate values for both water-use and nutrient-related variables. The second axis (PC2) explained 13.7% of the variance and was mostly related to traits associated with plant growth and canopy structure, such as LAI and shoot elongation (loading factors: 0.81 and 0.67, respectively; see Table A3). This axis captured seasonal shifts more than interspecific differences, as all species showed intra-annual displacement along PC2, while remaining relatively close to their species centroids along PC1.

## 3. Discussion

In recent years, the rapid expansion of *R. monosperma* has raised concerns about the conservation of the native coastal dune plant communities of Doñana National Park. This species does not exhibit a single, clearly distinct ecophysiological strategy that would by itself explain its success. However, it displays a mosaic of morphological and functional traits that likely contribute to its fast growth and competitive advantage. These traits reflect a physiological strategy common among species adapted to arid Mediterranean environments and nutrient-poor soils [42,50], characterised by drought tolerance and an efficient balance between gas exchange and water conservation. In particular, *R. monosperma* is distinguished from the other species by its ability to fix atmospheric nitrogen, resulting in significantly higher foliar nitrogen content; its greater photochemical efficiency during summer; and its high morpho-physiological plasticity, which allows seasonal adjustment of key traits for efficient resource use. Together, these attributes contribute to its competitive advantage and expansive behaviour.

The four study species exhibited a similar response to topography, suggesting that elevation does not impose a strong selective pressure in this system beyond its interaction with summer drought, as revealed by the repeated-measures ANOVA. Rather than promoting niche differentiation, the topographic gradient appears to act as a shared environmental constraint, with species adjusting key traits—such as LDMC, δ^15^N, shoot water potential, or photochemical efficiency—under increasing drought stress [51].

Regarding water-use strategies, *R. monosperma* appears to follow a more opportunistic approach, being able to rapidly rehydrate its branches just after the first rains in autumn, as reflected by the highest RWC. In contrast, *P. pinea* exhibited a more conservative strategy, maintaining relatively stable and elevated RWC values throughout the year, consistent with a more isohydric behaviour. Umbrella pines have been planted in the area since the 19th century, significantly affecting sand mobility, salt spray deposition, and the spatial distribution of vegetation [48]. In the extreme SE of the park, however, pine populations are now naturalised and stable, coexisting with native Juniperus species. This long-term presence and ecological integration of *P. pinea* may partly explain its functional convergence with native species in terms of water status regulation. The lowest RWC values were recorded in *J. phoenicea* during the summer of 2024, regardless of its topographic position, suggesting a limited capacity to buffer water loss during drought. Although the midday shoot water potential was measured only in summer, *J. phoenicea* reached the most negative values (especially in the higher topographic site), while the other three species maintained similar values close to −2 MPa, across the topographic position, reflecting more effective control of water status. This variable represents the balance between transpiration and water supply to the leaves via xylem conductivity [46], and our findings are consistent with previous studies in the stabilised dunes of Doñana, where *J. phoenicea* reached midday shoot water potentials below −9 MPa in late summer [37]. These traits may partly explain the higher drought vulnerability of *J. phoenicea* and raise questions about its long-term persistence under increasing aridity. In contrast, the better water status of *R. monosperma*, together with its high plasticity and other functional advantages, could contribute to its competitive advantage under increasing aridity.

The δ^13^C values provide an integrative variable of stomatal control and WUEi over the entire period of leaf development [52,53,54]. Our results indicate that *P. pinea*, particularly in lower topographic zones, always presents the most negative values. This result suggests that groundwater was always available for root uptake, thereby minimising the need for water-saving responses, and stomatal conductance remained high. In contrast, *J. phoenicea* displays the least negative δ^13^C values, which suggests higher water-use efficiency, consistent with its more negative midday shoot water potential. The lack of more pronounced seasonal differences in δ^13^C among species may reflect their particular growing strategies. As typical native Mediterranean species, they tend to restrict growth to periods of more favourable moisture conditions, so δ^13^C likely captures the physiological status during active growth periods, rather than during drought-stressful periods. Nevertheless, following [43], δ^13^C remains a robust functional indicator of interspecific differences in water-use strategies, especially when interpreted alongside other physiological variables.

The morpho-functional leaf traits of coastal dune species reflect adaptations to resource-poor environments, where plant productivity is strongly limited by water and nutrient availability [46,55,56]. In Mediterranean habitats, leaves tend to be small, tough, often hairy, and sometimes rolled dorsoventrally to form stomatal grooves, which reduce water loss and improve drought resistance [46]. The study species exhibit marked differences in leaf morphology: *J. macrocarpa* has small, rigid leaves; *J. phoenicea* bears scale-like leaves; *R. monosperma* is essentially leafless, relying instead on green twigs (cladodes) for photosynthesis; and *P. pinea* presents needle-like leaves. This morphological variability is reflected in the values of the LMA. Notably, *R. monosperma* showed significant seasonal differences. In winter and spring, the new twigs are softer and thinner, resulting in lower LMA, whereas in summer, they become lignified and display higher values. This seasonal plasticity likely represents a protective strategy for shielding plant photosynthetic organs from summer stress. The LDMC values in *R. monosperma* present a similar seasonal pattern, further supporting its morphological and physiological plasticity under Mediterranean climate constraints.

Shoot elongation peaked in spring, which corresponds to the optimal phenological period of growth in the Mediterranean climate. However, our results also show that elongation was significantly greater in the lower topographic zones, where water availability is higher. Similar results were found in *Halimium halimifolium* in the stabilised sands of Doñana, where shoot elongation and ramification length were significantly higher in the wet areas in comparison with the drier areas of the dunes [57]. The only exception was *J. phoenicea*, which did not present significant differences in shoot growth between topographic levels.

During summer, *R. monosperma* exhibited significantly higher photochemical efficiency than the other species, whereas no significant differences were observed during the rest of the year. This pattern was consistent across both summers of 2023 and 2024. These results suggest that *R. monosperma* displays a higher photosynthetic capacity during the summer in comparison with the other species, which might contribute to its expansive character. In another coastal area of SW Spain, *R. monosperma* is able to compete with the endangered *Thymus carnosus* for water uptake; the plants of *Thymus* growing under *Retama* have to shift the seasonal water sources in comparison with isolated *Thymus* plants [58].

Concerning nutrient traits, δ^15^N values clearly show differences in nitrogen uptake strategies among the study species. In *R. monosperma,* the values remained close to 0‰ throughout the year, consistent with atmospheric nitrogen inputs via N_2_ fixation, which is characteristic of this expansive leguminous species. In addition, *R. monosperma* shows the highest foliar nitrogen content, which likely contributes to increased nitrogen availability in surrounding soils and may influence the nutritional status of neighbouring plants, as observed in *Acacia longifolia* [59]. *Pinus pinea* consistently showed the most negative values, whereas *J. macrocarpa* exhibited the strongest seasonal differences. The interpretation of δ^15^N values in natural systems is often complex. More negative values, such as those found in *P. pinea*, may result from a variety of factors, including internal N recycling [41,60,61] or the presence of symbiotic associations with mycorrhizal or bacterial, which often discriminate against ^15^N [62]. For instance, Zunzunegui et al. [41] found a negative linear relationship between foliar δ^15^N and distance from the sea in coastal dune species, suggesting a gradual shift in the main N sources across the dune system.

The consistently low LAI values observed in *R. monosperma* reflect its sparse canopy architecture, a trait that may play a key role in shaping plant–plant interactions in dune ecosystems. Combined with its capacity to fertilise the soil with nitrogen, as has been shown in the invasive legume *Acacia longifolia* [59], this species may act as a facilitator by creating microhabitats more favourable for the recruitment of nitrophilous plants, such as fertility islands [39]. Such facilitation processes could drive significant shifts in the community structure, favouring more competitive or nutrient-demanding species and potentially reducing the dominance of oligotrophic specialists. Nevertheless, this facilitative effect is not without trade-offs; scientific evidence suggests that *R. monosperma* may negatively affect endangered coastal dune species, such as *T. carnosus* [36], or contribute to the transformation of coastal dunes into shrubby formations where nitrophilous and ruderal plant species proliferate [63], raising concerns about its expanding dominance in conserved dune systems.

PCA provides an integrative overview of the functional strategies of the four study species, synthesising the variation across morpho-functional traits and seasonal conditions. The analysis identified three distinct functional types. *Pinus pinea* is clearly separated along the negative end of axis I, associated with traits indicative of stable water relations, such as less negative shoot water potential, higher RWC, and greater WUEi (more negative δ^13^C), suggesting a conservative but efficient water-use strategy. In contrast, *R. monosperma* occupies the positive end of Axis I, reflecting traits linked to nitrogen enrichment (higher N content and δ^15^N), which indicate N uptake through atmospheric N_2_ fixation and influence nutrient dynamics in the system. Both *Juniperus* species lay in the centre of the ordination space, indicating more intermediate morpho-functional trait values, which may provide more stress tolerance to the coastal-sand ecosystem.

Notably, *R. monosperma* also presents a clear seasonal separation along axis II, reflecting its high morphological and physiological plasticity in response to Mediterranean seasonality, a feature that may partly explain its competitive and expansive behaviour.

The results reveal that *R. monosperma* benefits from enhanced nitrogen acquisition via atmospheric nitrogen fixation, a functional advantage in the nutrient-poor soils of coastal dunes that may support its expansion. However, beyond this trait, *R. monosperma* does not show markedly distinctive morpho-functional traits compared with the other coexisting species, perhaps reflecting its status as a native rather than an exotic invader. Unlike invasive non-native legumes such as *Acacia longifolia* in Portugal [46] or *Oenothera drummondii* in the nearby coastal dunes of Huelva [13]. *Retama monosperma* likely co-evolved with the regional flora and environmental constraints.

Nevertheless, the key to its expansive success may lie in its marked morphophysiological plasticity. The ability to adjust key traits across seasons appears to enable *R. monosperma* to use resources efficiently year-round, promoting its expansive character. As suggested by other authors, invasive species often have stronger environmental adaptability, thereby ensuring competitiveness for habitat resources [64]. In this context, the greater seasonal plasticity of *R. monosperma* may be the key to its capacity to grow, spread, and outcompete other native coastal dune species.

## 4. Materials and Methods

### 4.1. Study Site

**Figure A1:**
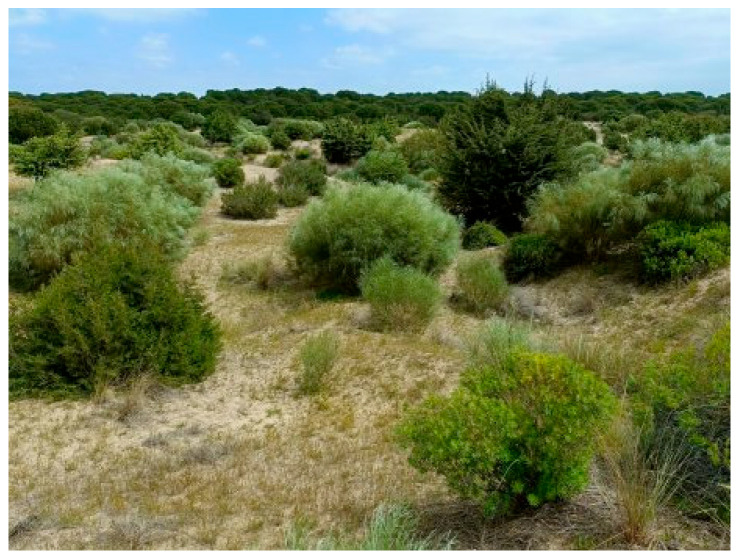
Coastal dune landscape at Doñana National Park (SW Spain), near the mouth of the Guadalquivir River (Huelva, 36.785° N, 6.396° W).

The study was conducted in a coastal dune system south of Doñana National Park (SW Spain), near the mouth of the Guadalquivir River (Huelva; ~36.785°N, 6.396°W). The area was newly created, formed over the last 80 years, as a result of beach progradation and the successive formation of dune ridges separated by depressed areas (Figure A1). The climate is Mediterranean sub-humid (Csa), with a mean annual precipitation of 523 mm and a pronounced summer drought, as most rainfall occurs between October and May. The mean annual temperature was 18.7 °C, ranging from 11.7 °C in the coldest month to 26.5 °C in the warmest month. As the area has formed, a process of primary succession has taken place and continues to be active. The woody vegetation is dominated by the shrub *R. monosperma* and the tree species *P. pinea*, *J. phoenicea*, and *J. macrocarpa*. The ecological functioning of these dune systems is strongly influenced by groundwater depth, seasonal drought, and anthropogenic pressures [65,66,67], making them particularly vulnerable to climate change and hydrological alterations.

**Figure 8:**
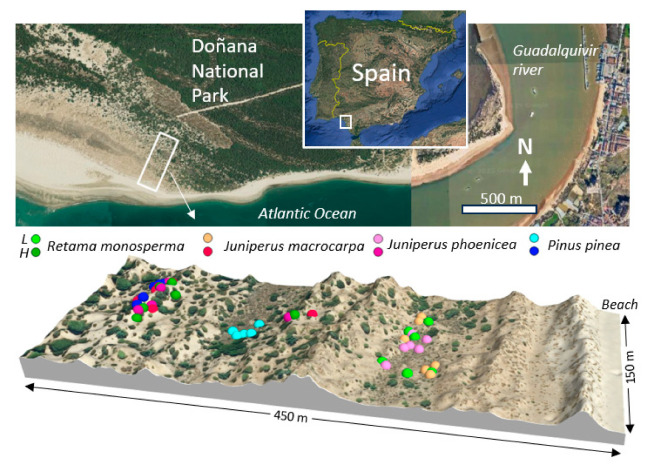
Aerial image of the study site showing the location of all marked individuals on the topography of the study area. Letters indicate the zones: L for low zone and H for high zone.

**Figure 9:**
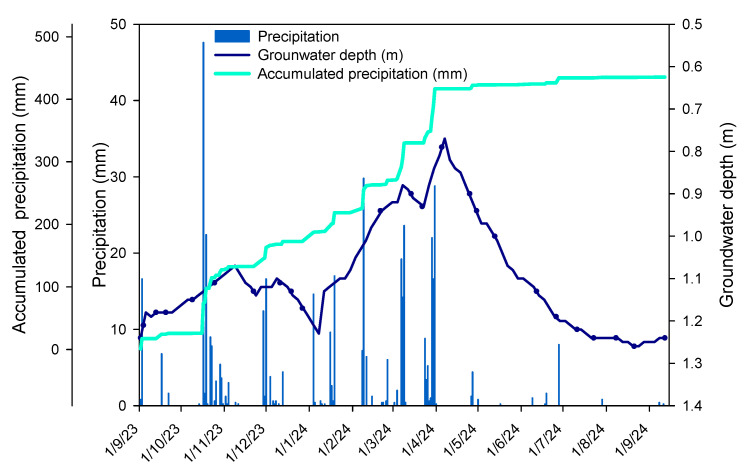
Daily and accumulated precipitation and groundwater depth in the low zone throughout the study period.

Two study zones with contrasting water availability were selected within the coastal dune system (Figure 8): a low zone, located behind the first and second dune ridges and closer to the water table (0.90 m during the humid period to 1.26 m in summer; Figure 9), and a high zone, situated on the third dune ridge, where groundwater is substantially deeper (4 to 6 m). At each site, six healthy, adult, and isolated individuals of each species (*R. monosperma*, *J. phoenicea*, *J. macrocarpa*, and *P. pinea*) were tagged and monitored seasonally from September 2023 to September 2024: A total of 48 individuals were studied (4 species × 2 zones × 6 individuals), ensuring consistent physiological measurements across the study period.

### 4.2. Study Species

**Figure A2:**
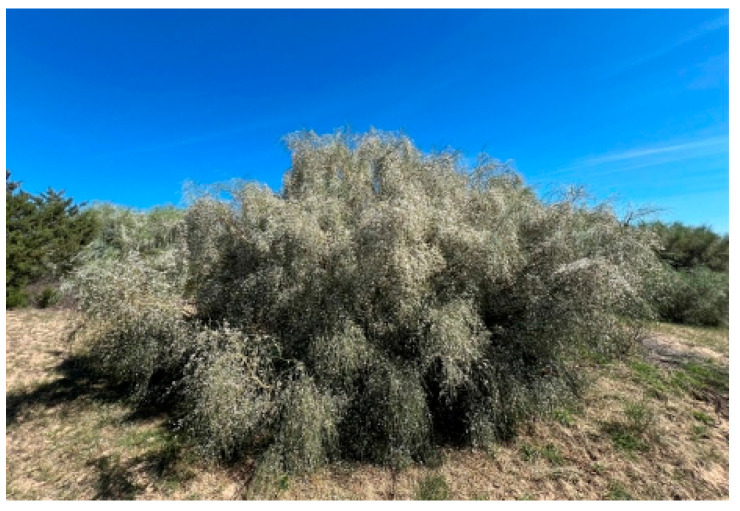
*Retama monosperma* flowering adult in summer.

*Retama monosperma* (L.) Boiss (Fabaceae) is a leafless, nitrogen-fixing shrub that can reach up to 7 m in height (Figure A2). Its distribution is restricted to the sandy coastal areas of the south-western Iberian Peninsula and north-western Morocco. The species flowers in winter (January–March) and has a high reproductive output, producing single-seeded legumes that, once ripe, fall to the ground between June and September [68]. It is a salinity-tolerant species [40], and its roots reach the dune groundwater [44].

**Figure A3:**
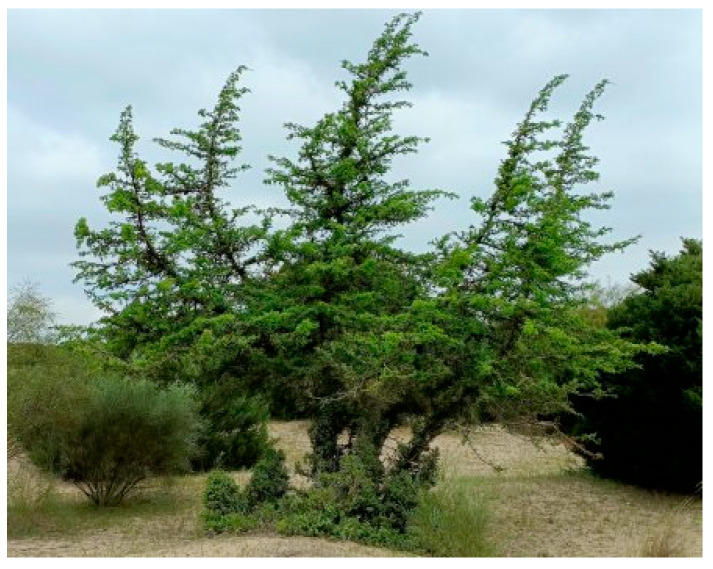
Adult individual of *Juniperus macrocarpa* along with co-occurring species *Juniperus phoenicea* and the scrub *Retama monosperma.*

**Figure A4:**
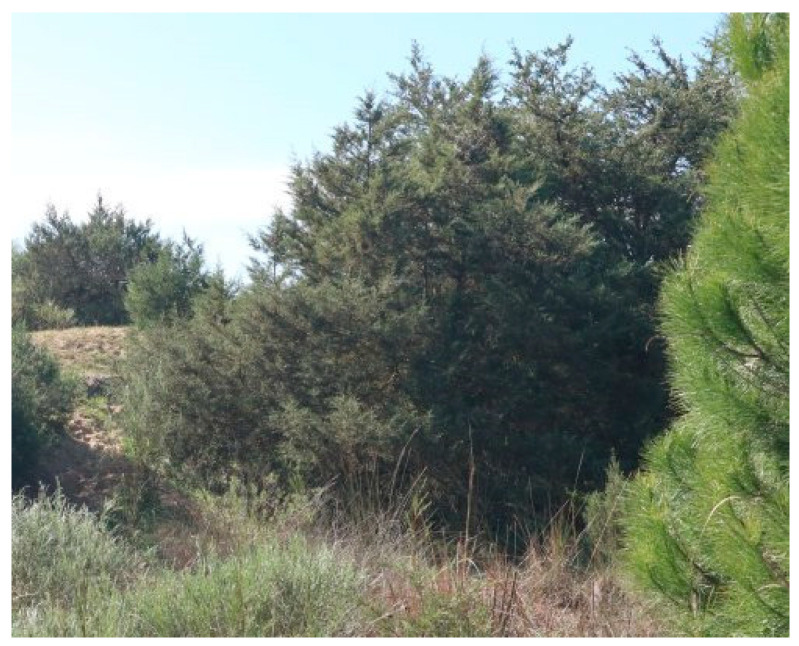
Adult *Juniperus phoenicea* individual along with the co-occurring species *Pinus pinea.*

*Juniperus macrocarpa* and *J. phoenicea* L. (Cupressaceae) are evergreen, slow-growing, dioecious shrubs or small trees (1–8 m), typically associated with xeric coastal habitats across the Mediterranean basin (Figure A3 and Figure A4). Their berry-like seed cones take more than a year to mature and are dispersed primarily by endozoochory and barochory [69,70,71,72]. Natural regeneration in both taxa is limited by factors such as low seed viability, poor germination success, and slow growth rate, which contribute to their fragmented distribution [73,74]. Both species are key structural components of coastal woodland communities and are considered indicators of mature dune succession stages. However, they are particularly vulnerable to habitat fragmentation and anthropogenic pressures.

**Figure A5:**
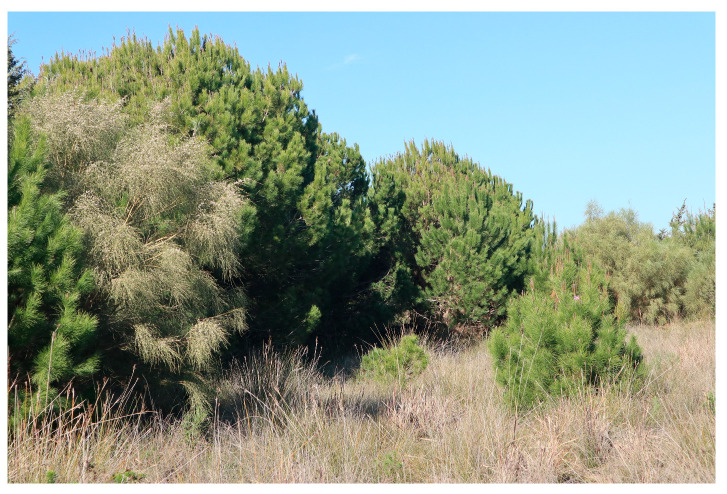
Adult *Pinus pinea* individuals at the low site, with a *Retama monosperma* plant growing *among the pines.*

*Pinus pinea* L. *(Pinaceae)* is a long-lived, anemophilous conifer commonly planted or naturally regenerating in dune systems [48,75]. Although it can reach 12 m in height, its colonisation rate on mobile coastal dunes is relatively slow compared to the other species (Figure A5). *P. pinea* has been planted because it stabilises the dunes, modifies the microclimate beneath its canopy, and reduces harsh abiotic conditions in coastal dunes through its own structure [48,76]. Despite being widely naturalised, *P. pinea* is not considered invasive in most regions, although its dense canopy and litter accumulation can alter dune vegetation dynamics.

All four species play key roles in community succession and dune stabilisation, but differ markedly in life history traits and colonisation dynamics.

### 4.3. Methodology

To evaluate whether *R. monosperma* exhibits ecophysiological traits that confer a growth advantage over *J. phoenicea*, *J. macrocarpa*, and *P. pinea*, we measured seasonal variations in several physiological and structural variables in adult individuals of the four species under two water availability levels, with a seasonal sampling frequency. In total, 48 marked individuals were measured (6 plants × 2 sites × 4 species). The following measurements were conducted on each marked individual:

#### 4.3.1. Individual Size Measurements

Plant height, as well as maximum and minimum crown diameter, were recorded at the beginning of the study period.

#### 4.3.2. Leaf Area Index (LAI, m^2^/m^2^)

The LAI was estimated using an AccuPAR LP-80 ceptometer, which calculates the LAI based on the attenuation of photosynthetically active radiation (400–700 nm) through the canopy. For each individual, two external readings and 5 to 10 below-canopy measurements were taken at the ground level around the crown.

#### 4.3.3. Shoot Elongation and Brach Number

At the end of the growing season, the annual shoot elongation was assessed for each marked individual. Ten branches per individual were randomly selected, and the length of the current year’s growth was measured from the terminal bud scar to the shoot apex using a digital calliper.

#### 4.3.4. Photochemical Efficiency (Φ_PSII_) and Stomatal Conductance (g_s_)

The photochemical efficiency and stomatal conductance were measured under ambient light conditions using an LI-600 porometer-fluorometer (LI-COR Biosciences, Lincoln, NE, USA). Five sun-exposed, photosynthetically active units (leaves, cladodes, or needles, depending on the species) were measured per individual, ensuring consistent sampling across species and sites.

#### 4.3.5. Leaf and Cladode Isotopic Analysis and N and C Content

Stable isotope analysis of carbon and nitrogen: Leaf samples were collected from each marked individual, dried at 65 °C for 48 h until constant weight, and finely ground using a ball mill. The resulting powder was used to determine the carbon and nitrogen stable isotope composition (δ^13^C and δ^15^N). Isotopic analyses were performed using an elemental analyser (Leco CHNS-932, Madrid, Spain) coupled to a continuous flow isotope ratio mass spectrometer (Thermo Scientific Delta V spectrometer, Waltham, MA, USA). The isotope ratios of ^13^C/^12^C and ^15^N/^14^N were expressed in delta (δ) notation in per mil (‰) relative to international standards (VPDB for δ^13^C and atmospheric N_2_ for δ^15^N). The values were calculated using the following equation:

δ (‰) = 1000 × (R_sample_ − R_standard_)/R_standard_ (1)

where R represents the isotope ratio (^13^C/^12^C or ^15^N/^14^N) in the samples and the corresponding international standards.

Although δ^15^N values in plant tissues do not directly mirror the isotopic signature of nitrogen sources due to isotopic discrimination during uptake and internal processing [77,78,79], this discrimination is generally minimal in nutrient-poor environments such as Mediterranean coastal dunes. Therefore, foliar δ^15^N can provide valuable insights into nitrogen inputs and cycling within these ecosystems [80,81,82].

Foliar δ^13^C was used as a proxy for long-term intrinsic water-use efficiency (WUEi), based on the relationship between stomatal regulation, internal CO_2_ concentration, and discrimination against the heavier carbon isotope during photosynthesis [52,53,54]. Greater stomatal closure reduces discrimination against ^13^C, leading to more enriched δ^13^C values and increased WUEi.

In addition to the isotopic ratios, the total nitrogen and carbon content (%N, %C) of the April 2024 samples were quantified using the same elemental analyser setup.

#### 4.3.6. Midday Shoot Water Potential (Ψm)

The plant water status was assessed at midday (12:30–14:30 solar time) in September 2024, when the minimum water potential typically occurs. The terminal shoots were excised and immediately placed in unsealed, transparent plastic bags wrapped in a moist paper towel to maintain high humidity. These bags were then enclosed in larger, sealed, opaque zip-lock bags that had been pre-exhaled into, further minimising water loss and protecting the sample from light exposure [83]. The samples were transported in a portable icebox (ice not in contact with the plant material) and measured 3–5 h after collection using a pressure chamber (Manofrígido, Lisbon, Portugal) in the laboratory.

#### 4.3.7. Leaf Traits

Fully expanded, healthy leaves and twigs (~10 g) were collected from each of the 48 marked individuals. The samples were stored in plastic bags under cold conditions, and the fresh mass (Mf) was recorded within 3 h of collection. Measurements were conducted seasonally and included the following:

- Relative water content (RWC, %): The leaf and stem water content was determined by weighing fresh leaves upon arrival at the laboratory (Mf). The leaves were then rehydrated in distilled water for 24 h at 5 °C to obtain their turgid mass (Mt) after gently blotting them dry. Finally, they were oven-dried at 70 °C for 48 h to obtain the dry mass (Md). RWC was calculated as follows:
  RWC% = (Mf − Md)/(Mt − Md) × 100(2)
- Leaf dry matter content (LDMC, mg g^−1^): LDMC was calculated using the same samples as for RWC, as follows:
  LDMC = Md/Mt(3)
- Leaf mass per area (LMA, g m^−2^): Leaf area was estimated using the mobile application Easy Leaf Area, which calculates the surface area by comparison with a known reference. The leaves were then oven-dried at 70 °C for 48 h and weighed; LMA was calculated as follows:
  LMA = Md/Leaf area(4)

### 4.4. Statistical Analysis

To evaluate the differences in the physiological and structural variables of each species in each zone measured throughout the year, we conducted a three-way repeated-measure analysis of variance (ANOVA), in which the within-subject factor was time (with five categories) and the between-subject factors were water availability and species. In addition, one-way ANOVA was performed to evaluate differences between species at each measurement date. When significant effects were found, post hoc pairwise comparisons were carried out using Tukey’s HSD test. Due to the non-normal distribution of the shoot elongation data, this variable was analysed using the non-parametric Kruskal–Wallis test, followed by Mann–Whitney U tests for pairwise comparisons.

A principal component analysis (PCA) was performed to explore the multivariate patterns and visualise the overall differences in the ecophysiological response among species across seasons and groundwater levels. Before PCA, the variables were standardised to ensure comparability.

In addition, linear regression analysis was used to examine the relationships between selected functional traits (e.g., shoot elongation, leaf water content, and photochemical efficiency) and environmental variables (e.g., groundwater availability or season).

The normality of the data was tested using the Kolmogorov–Smirnov test. All statistical analyses were performed using R software (version 4.4.2, R Core Team, 2025). A significance level of *p* < 0.05 was used throughout all statistical tests.

## 5. Conclusions

Coastal dune systems have been transformed and urbanised in many areas of the world. Well-preserved dunes, such as the mobile dune system of Doñana National Park, represent a unique system that should be managed with careful strategies.

Our results show that the expansive success of the native species *R. monosperma* in Mediterranean coastal dunes is linked to a combination of functional traits. Its ability to fix atmospheric nitrogen, along with its high morphological and physiological plasticity, contributes to its competitive advantage. In particular, its higher summer photochemical efficiency and rapid rehydration after autumn rains enhance its performance under harsh dune conditions. These traits may promote its expansion and alter dune community structure and function, highlighting the need to consider the role of native-expansive species in conservation strategies for threatened coastal dune ecosystems.

Moreover, the comparative analysis reveals that the co-occurring species exhibit distinct functional strategies. *Pinus pinea* shows a conservative but efficient water-use strategy, with a stable water status and higher water-use efficiency, likely favoured by deeper root access. In contrast, both *Juniperus* species displayed more intermediate morpho-functional values, possibly reflecting greater stress tolerance but lower responsiveness to seasonal fluctuations. This functional diversity among coexisting woody species illustrates contrasting adaptive strategies in dune systems and helps explain the differential success of *R. monosperma* under current environmental pressures.

## Data Availability

Data will be made available upon request.

## Abbreviations

The following abbreviations are used in this manuscript:

RWC	Relative water content
Ψ_m_	Midday shoot water potential
Φ_PSII_	Effective photochemical efficiency
g_s_	Stomatal conductance
LAI	Leaf area index
LMA	Leaf mass area
LDMC	Leaf dry matter content
%N	Total nitrogen content
%C	Total carbon content
δ^15^N	Nitrogen isotope signature (^15^N/^14^N)
δ^13^C	Carbon isotope signature (^13^C/^12^C)
WUEi	Long-term intrinsic water-use efficiency

## Appendix A

### Results of the Statistical Analysis

**Table A1:** Results of the repeated measures ANOVA for the factors season (Time, T), species, and topography (Zone, Z) across the five sampling campaigns conducted during the study period on the variables shoot elongation (Growth), relative water content (RWC), leaf dry mass area (LMA), leaf dry matter content (LDMC), effective photochemical efficiency (Φ_PSII_), LAI (leaf area index), nitrogen and carbon isotopic ratio (δ^15^N and δ^13^C), and two-way ANOVA for shoot water potential (Ψ_m_). Significant differences (*p* < 0.05) are highlighted in bold. Variables that did not meet the assumptions of ANOVA were analysed using non-parametric Kruskal–Wallis tests (Table A2).

**Within-Subject Effects**												
	**Time**			**Time × Zone**			**Time × Species**			**T × Species × Z**		
	df	F	*p*	df	F	*p*	df	F	*p*	df	F	*p*
RWC	4	64.29	**<0.001**	4	1.261	0.288	12	8.85	**<0.001**	12.0	1.10	0.357
LMA	3.1	22.14	**<0.001**	3.10	1.437	0.234	9.3	30.90	**<0.001**	9.3	3.13	**0.002**
LDMC	8.2	341.21	**<0.001**	2.73	6.309	**0.001**	8.2	41.91	**<0.001**	8.2	6.50	**<0.0001**
Growth	2.0	164.12	**<0.001**	2	7.350	**0.001**	6	12.41	**<0.001**	6.0	3.37	**0.005**
LAI	2.4	139.96	**<0.001**	2.48	12.572	**<0.001**	7.4	8.439	**<0.001**	7.4	0.99	0.446
Φ_PSI_	2.6	19.94	**<0.001**	2.64	8.630	**0.005**	7.9	9.007	**<0.001**	7.9	5.51	**0.016**
δ^13^C	1.0	34.78	**<0.001**	1.04	1.954	0.169	3.1	0.978	0.415	3.1	0.89	0.454
δ^15^N	3.0	259.08	**<0.001**	3	0.750	0.524	9	26.57	**<0.001**	9.0	5.46	**<0.0001**
Between-subject effects												
	**Species**			**Zone**			**Zone × Specie**					
	df	F	*p*	df	F	*p*	df	F	*p*			
RWC	3	10.72	**<0.001**	1	4.20	**0.047**	3	0.97	0.414			
LMA	3	213.63	**<0.001**	1	4.84	**0.034**	3	0.16	0.922			
LDMC	3	370.85	**<0.001**	1	109.84	**<0.001**	3	14.93	**<0.001**			
Growth	3	31.57	**<0.001**	1	82.06	**<0.001**	3	2.74	0.056			
LAI	3	7.98	**<0.001**	1	1.30	0.259	3	3.36	**0.028**			
Fv/Fm	3	26.09	**<0.001**	1	3.28	0.078	3	1.54	0.219			
δ^13^C	3	10.64	**<0.001**	1	1.86	0.179	3	2.55	0.069			
δ^15^N	3	91.81	**<0.001**	1	7.82	**0.008**	3	1.02	0.390			
Ψ_m_	3	20,73	**<0.001**	1	23.24	**<0.001**	3	4.905	**0.005**			

**Table A2:** Results of the Kruskal–Wallis tests for variables that did not meet the assumptions of parametric analysis on the variables: leaf N and C content (%N, %C) and stomatal conductance (g_s_). Significant differences (*p* < 0.05) are highlighted in bold.

l	Zone			Species			Time		
	df	H	***p***	df	H	***p***	df	H	***p***
% C	1	4.76	**0.029**	3	5.92	<0.116	1	0.87	0.35
%N	1	0.75	0.387	3	53.56	**<0.001**	1	19.78	**<0.001**
g_s_	1	0.61	0.433	3	10.72	**0.013**	1	24.10	**<0.001**

**Table A3:** Mean values (± SD) of leaf area index (LAI, m^2^ m^−2^), leaf mass per area (LMA, g m^−2^), and leaf dry matter content (LDMC, mg g^−1^) across the five sampling campaigns for the four study species.

Species	Season	LAI ± sd		LMA ± sd		LDMC ± sd	
*Juniperus macrocarpa*	SEPTEMBER-23			217	±22	461	±30.8
	APRIL-24	6.7	±2.1	163	±8	353	±26.4
	DECEMBER-23	1.6	±0.5	429	±83	388	±80.5
	FEBRUARY-24	2.9	±1.3	246	±17	470	±37.7
	SEPTEMBER-24	2.1	±1.5	226	±30	465	±58.6
*Pinus pinea*	SEPTEMBER-23			236	±24	358	±19.2
	APRIL-24	3.4	±1.5	233	±53	237	±19.0
	DECEMBER-23	0.9	±0.6	173	±34	232	±17.7
	FEBRUARY-24	1.0	±0.7	188	±18	224	±14.8
	SEPTEMBER-24	1.1	0.6	439	±84	443	±38.1
*Retama monosperma*	SEPTEMBER-23			458	±57	429	±33.5
	APRIL-24	5.1	±1.5	396	±47	395	±38.4
	DECEMBER-23	2.1	±1.2	440	±58	411	±34.3
	FEBRUARY-24	1.1	±1.0	405	±43	405	±35.5
	SEPTEMBER-24	1.7	1.2	474	±78	544	±28.6
*Juniperus phoenicia*	SEPTEMBER-23			366	±29	483	±17.5
	APRIL-24	6.3	±1.8	349	±30	379	±18.2
	DECEMBER-23	1.9	±1.1	292	±34	363	±18.7
	FEBRUARY-24	2.3	±1.4	298	±37	369	±20.9
	SEPTEMBER-24	2.5	±1.6	241	±22	383	±19.4

**Table A4:** Variable loadings on the first two principal components (PC1 and PC2) from the PCA conducted on morphological and physiological traits measured in the four study species throughout the year. Values indicate the correlation (loading) between each variable and the principal components. Shoot elongation (Growth), relative water content (RWC), leaf dry mass area (LMA), leaf dry matter content (LDMC), effective photochemical efficiency (Φ_PSII_), leaf area index (LAI), and nitrogen and carbon stable isotope ratios (δ^15^N and δ^13^C). Bold values highlight variables with high contributions to each PC axis.

Variable	PC1	PC2
δ^15^N	**0.45**	−0.11
WUEi	0.35	0.134
**% N**	**0.51**	0.130
% C	−0.33	−0.151
LAI	0.18	**0.81**
RWC	**−0.43**	−0.15
LMA	**0.77**	−0.34
LDMC	**0.73**	−0.17
Growth	0.37	**0.68**
Φ_PSII_	0.29	**−0.49**
Ψ_m_	**−0.43**	0.20
gs	−0.29	−0.13
